# Metabolomic changes in preterminal serum samples of rhesus macaques exposed to two different lethal doses of total-body gamma-radiation

**DOI:** 10.1038/s41598-024-75225-3

**Published:** 2024-10-13

**Authors:** Alana D. Carpenter, Keirstyn M. Empfield, Sarah A. Petrus, Oluseyi O. Fatanmi, Stephen Y. Wise, John B. Tyburski, Amrita K. Cheema, Vijay K. Singh

**Affiliations:** 1 Division of Radioprotectants, Department of Pharmacology and Molecular Therapeutics, F. Edward Hébert School of Medicine, Uniformed Services University of the Health Sciences, Bethesda, MD 20814 USA; 2 Armed Forces Radiobiology Research Institute, Uniformed Services University of the Health Sciences, 4301, Jones Bridge Road, Bethesda, MD 20814 USA; 3 Nelson Scientific Labs, LLC, Potomac, MD 20854 USA; 4 Department of Oncology, Lombardi Comprehensive Cancer Center, Georgetown University Medical Center, Washington, DC 20057 USA; 5 Department of Biochemistry, Molecular and Cellular Biology, Georgetown University Medical Center, Washington, DC 20057 USA

**Keywords:** Biomarkers, Metabolomics

## Abstract

Exposure to ionizing radiation induces cellular and molecular damage leading to a cascade of events resulting in tissue and organ injury. Our study strives to characterize and validate metabolomic changes in preterminal stage (immediately prior to death) samples collected from rhesus macaques lethally irradiated with one of two different doses of radiation. Peripheral blood samples were collected pre-exposure, post-exposure, and at the preterminal stage of nonhuman primates (NHPs that did not survive exposure with 7.2 Gy or 7.6 Gy total-body radiation (LD_60-80/60_)). We analyzed global metabolomic alterations using ultra-performance liquid chromatography (UPLC) quadrupole time-of-flight mass spectrometry (QTOF-MS) in serum samples collected at various timepoints in relation to radiation exposure. The goal of this study was to validate the metabolic shifts present in samples collected just prior to death, which were reported earlier in a preliminary study with a limited number of samples and a single dose of radiation. Here, we demonstrate that radiation exposure induced significant time-dependent metabolic alterations compared with pre-exposure samples. We observed significant metabolite dysregulation in animals exposed to 7.6 Gy compared to 7.2 Gy. Greater metabolic disruption was observed in the preterminal groups than all of the other post-irradiation timepoints in both cohorts. Metabolomic shifts in these preterminal groups also revealed consistent disturbances in sphingolipid metabolism, steroid hormone biosynthesis, and glycerophospholipid metabolism pathways. Overall, the sphingolipid metabolism pathway appears to be representative of the preterminal phenotype, confirming the results of our preliminary study. These results offer important and novel insights for identification and validation of biomarkers for lethality, and such observations would be valuable for triage during a radiological/nuclear mass casualty scenario.

## Introduction

Exposure to ionizing radiation damages cells and tissues. Though radiation exposure can directly damage DNA, it can also induce indirect damage to both DNA and other molecules including macromolecules found in cell membranes by way of the production of reactive oxygen species (ROS). The resulting injury can lead to altered cell function and cell death due to apoptosis. Furthermore, ROS can stimulate autophagy, the self-digestive progression that degrades cellular machineries^1,2^. This can lead to a positive effect due to the removal of damaged cells. The sensitivity of specific cell types to radiation exposure is directly related to its mitotic rate and indirectly related to its differentiated stage. Thus, cells of the lymphohematopoietic system, gametocytes, and the lining of the gastrointestinal (GI) tract are specifically susceptible to the effects of irradiation. On the other hand, non-dividing and highly differentiated cells of the central nervous system are comparatively resistant to the detrimental effects of radiation exposure.

Treating radiation-exposed victims requires an estimate of the received radiation dose^3^. One method of determining this is through the victim’s symptomology, where the Medical Treatment Protocols (METREPOL) system for radiation exposed individual may provide a triage scheme^4–6^. The METREPOL system serves as a foundation for a computerized guidance system to assess victims within the first 48 h of radiation exposure by considering various clinical and biological criteria (e.g., intensity of nausea, frequency of diarrhea, blood pressure, etc.). Although this can be useful for small to medium sized radiological accidents, the METREPOL system was not developed to be used during a large-scale event. Another option for estimating exposure is by using commercial dosimeters, but they come with several limitations. These dosimeters can only provide an accurate dose found in the surrounding air; they do not provide information for the radiation dose received. Furthermore, the likelihood that a dosimeter is present at the location of many radiological/nuclear events is very low.

Currently, the clinical strategy for evaluating radiation exposure is based on assessing clinical symptoms developed over time. Conventional biodosimetry techniques of monitoring clinical prodromal symptoms are not effective for individuals exposed to less than acute doses of ionizing radiation or to radiation exposures delivered more chronically. Furthermore, these methods are time-consuming and inconvenient for real-time, mass exposure scenarios^7^. Additionally, there is a several days-long latent period between radiation exposure and symptom presentation. During this time, it is critical to begin therapeutic interventions that could save the victim’s life. However, deciding on a therapeutic intervention can be difficult for medical providers if they have limited information on the level of radiation to which the patient has been exposed. Developing biochemical or molecular biomarkers could overcome this gap of knowledge and allow providers to deploy medical countermeasures (MCMs) in a timely manner. This would allow for a more effective triage and treatment^8,9^. A biomarker is a quantifiable biological feature that can indicate a specific biological, therapeutic or pathological value. In the context of radiation injury, biomarkers include blood cell counts, receptor expression, metabolites, lipidomes, microbiota, growth factors, cytokines, proteins, citrulline, tooth enamel and fingernail-based markers, chromosomal aberrations, gene sequences, miRNA, messenger RNA, long non-coding RNAs, and other imaging-based investigations^10^. A majority of the techniques are labor intensive, time consuming, require well-trained trained personnel, which is compounded by difficulty in obtain accurate dosimetry data.

Metabolomics is an emerging area that provides a snapshot of the expression of metabolites from several known pathways in response to exogenous stimuli. Metabolites play a vital role in human health and well-being. These molecules are outcomes of cascades of proteomic and genomic processes, making them perfect biomarkers for injury or disease progression^11–13^. Metabolite biomarkers are also valuable due to their easy identification and validation in biological samples, which can be conveniently collected without invasive techniques. Both untargeted (global) and targeted (quantitative phase) metabolomics have been used in various settings of radiation injuries and MCM development^12^.

The objective of this study was to validate biomarkers for 7.2 Gy radiation-induced moribundity in NHPs reported in an earlier metabolomics study. In the earlier study, we reported our preliminary observation using preterminal samples collected immediately prior to euthanasia of moribund animals at timepoints ranging from days 14 to 22 d post-irradiation. These preterminal samples showed a greater degree of metabolic dysregulation in comparison to all other timepoints tested^14^. Specifically, glycerophospholipid metabolism and the steroid hormone biosynthesis pathways were found to be significantly dysregulated in preterminal NHPs. Glycerophospholipid metabolism was found to be dysregulated both in post-irradiation and preterminal (moribund animal just prior to euthanasia) samples. This pathway is of particular interest as radiation is known to induce lipid peroxidation and dyslipidemia resulting in cellular damage^15^. In our current study, we examined the metabolites in peripheral blood serum samples collected from a different cohort of NHPs following exposure to 7.2 and 7.6 Gy (n = 10 in each group) total-body ^60^Co gamma-radiation (LD_60-80/60_) to validate the dysregulated metabolite profiles which differed from the samples collected at pre-exposure and early post-exposure timepoints. We were able to validate the results of our preliminary study demonstrating that lipid metabolism pathways (specifically, the glycerophospholipid metabolism pathway in the 7.2 Gy study, and the sphingolipid metabolism pathway in this current study) as well as the steroid hormone biosynthesis pathway were significantly dysregulated by both radiation doses, confirming that these metabolic pathways are particularly sensitive to ionizing radiation and early dysregulation in these pathways can be used as a readout of radiation induced adverse outcomes. Furthermore, as animals approached death, an increased expression in these pathways was found, indicating that radiation-induced dysregulation to these pathways is representative of the preterminal phenotype. These observations are of special significance because these metabolomic changes may aid in the identification of metabolites that can be used as biomarkers for triage after radiological mass casualty scenarios.

## Materials and Methods

### Experimental design

**Fig. 1:**
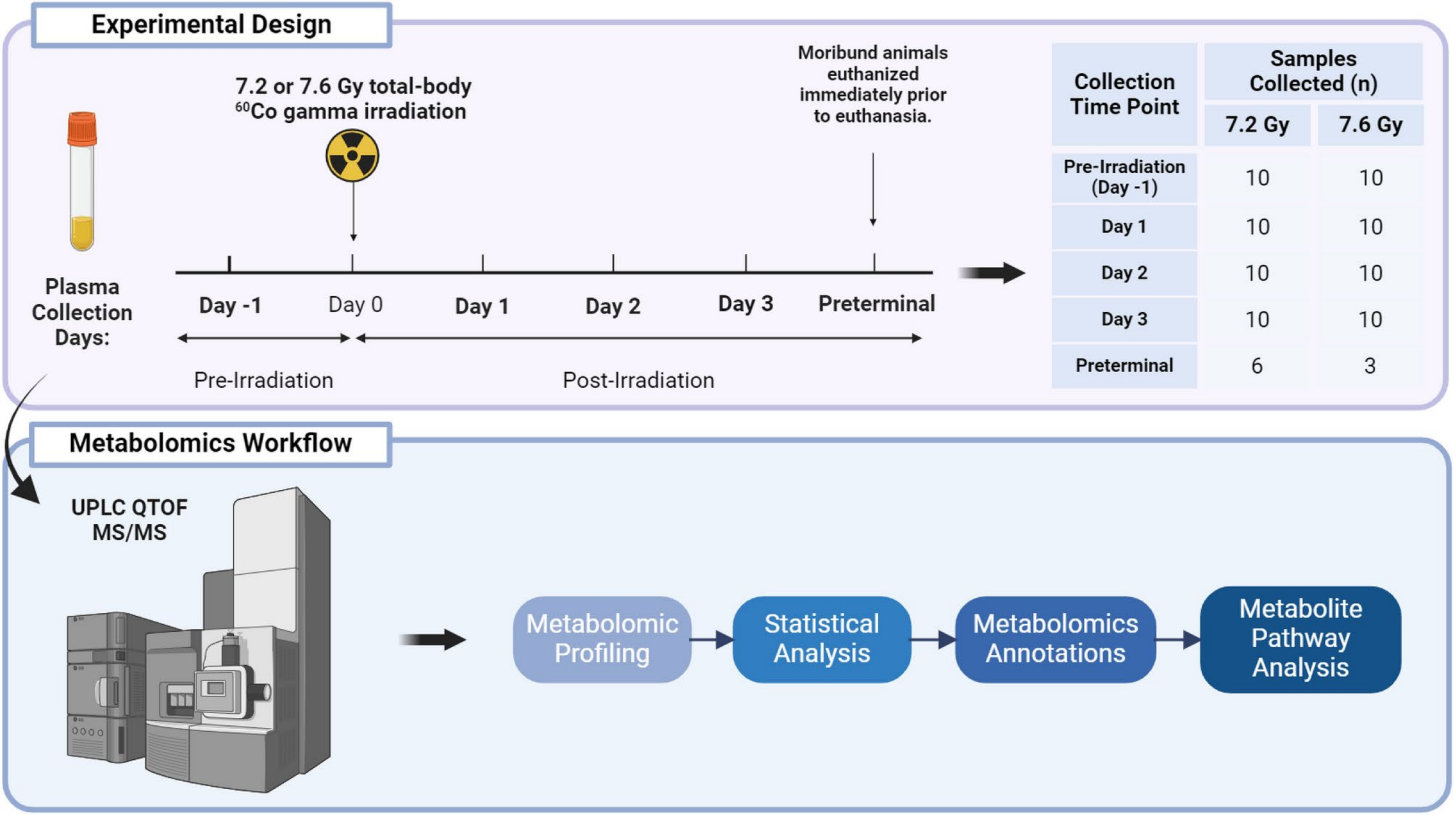
Overall study design for assessing the impact of radiation on serum metabolomics in NHPs exposed to 7.2 Gy or 7.6 Gy total-body irradiation. Samples were collected pre-irradiation, post-irradiation (on the following three days), and immediately prior to death (preterminal).

The goal of this study was to investigate the metabolic effects of 7.2 or 7.6 Gy total-body exposed NHP serum collected pre- and post-irradiation. Of particular interest were the preterminal samples collected from moribund animals. The preterminal samples were compared to other samples collected prior to irradiation as well as samples collected at various times points after irradiation. The timepoint and sample details can be seen below in Fig. 1.

### Animals

A total of 20 male NHPs (*Macaca mulatta*, Chinese sub-strain), 3.1 – 5.8 years of age, weighing between 4.5 – 7.0 kg were obtained from the National Institutes of Health Animal Center in Poolesville, MD. The Armed Forces Radiobiology Research Institute (AFRRI), an AAALAC International accredited facility, housed these animals. NHPs were quarantined for seven weeks prior to the initiation of this study. To prevent the possibility of conflict and subsequent injuries, all NHPs were housed individually with cage dividers to still allow social interaction. Animals were provided with a primate diet (Teklad T.2050 diet; Harlan Laboratories Inc., Madison, WI, USA) twice daily, with 4 biscuits each, at 7:00 AM and 2:00 PM. Drinking water was provided ad libitum^16^. The study design and procedures received approvals from the Institutional Animal Care and Use Committee of the Armed Forces Radiobiology Research Institute, Uniformed Services University of the Health Sciences and the Department of Defense Animal Care and Use Review Office (ACURO). All animal procedures were performed according to the *Guide for the Care and Use of Laboratory Animals* of the Institute of Laboratory Animal Resources, National Research Council, U.S. National Academy of Sciences^17^. This study is reported in accordance with ARRIVE guidelines.

### Irradiation

Approximately one week before irradiation, the NHPs’ abdominal lateral separations were measured using a digital caliper. Measurements were taken at the height of the dose prescription point (core of the abdomen). Animals were assigned to pairs based on the similarity of their lateral separations (+ /- 1 cm), and those who could not be matched within 1 cm of another animal’s measurements were irradiated independently. To prepare for the irradiation procedure, food was withheld from the NHPs 12–18 h before to minimize irradiation or sedation-induced vomiting. NHPs were safely restrained using the squeeze-back mechanism in their cage and 10 – 15 mg/kg of ketamine hydrochloride (100 mg/ml) was administered intramuscularly (*im*) roughly 15 – 30 min prior to the procedure. Once fully sedated, the animals were secured via restraints in a seated position inside custom-made Plexiglas irradiation boxes. The Plexiglas boxes were then transported via a HEPA filtered cart to the high-level cobalt-60 radiation facility. Here, each pair of NHPs were positioned on the irradiation platform facing in opposite directions. If needed, the animals were administered a ketamine booster (0.1 – 0.3 ml *im*) to ensure that the proper anesthetic plane was maintained throughout the procedure. The animals were then exposed to cobalt-60 total-body gamma radiation at a dose rate of 0.6 Gy/min. Ten NHPs were exposed to a total dose of 7.2 Gy, while the other 10 were irradiated with a dose of 7.6 Gy. All animals were observed during the procedure using an in-room video system. Upon completion, animals were returned to their cages in the vivarium using the transport cart. The animals were monitored by staff members until they recovered from sedation. Radiation exposures began at 8:00 AM and continued until about 12:00 noon. Additional details of total-body irradiation and dosimetry are given in earlier publications^18,19^.

### Cage-side animal observations

Cage-side animal observations were performed at least twice a day in the morning and afternoon for the quarantine and study periods. Beginning on the day of irradiation, clinical observations were recorded. Between days 10 to 20 post-irradiation (known as the critical period), animals were observed three times a day between 8 and 10 h apart. Animals that were deemed moribund using criteria listed on the approved protocol were scheduled for euthanasia based on the veterinarian’s discretion. Several parameters were used as guidelines for moribundity including inappetence, minimal or no response to stimuli, severe anemia, weakness, etc. An on-call veterinary technician/veterinarian was available 24 h a day in the event of an emergency situation^20^.

### Blood sample collection

Serum samples were enriched from whole blood collected on days -1 (pre-irradiation), 1, 2, 3, or immediately prior to euthanasia (preterminal). Blood was collected from a peripheral vessel (either saphenous or cephalic vein) as described earlier. If this route was unsuccessful due to the moribundity of the animal or other compromising injuries, the femoral vein was used as a secondary vessel for blood collection. The desired volume of blood was collected with a 3 ml disposable luer-lock syringe with a 25-gauge needle. The blood was placed in serum separating tubes and allowed to clot for at least 30 min prior to being centrifuged (10 min, 400 × g). The resulting serum was initially stored at -80 °C and later shipped to Georgetown University, Washington, DC, for metabolomic analysis.

### Euthanasia

Although the study period was scheduled for 60 days, many animals became moribund as a result of the radiation doses used (7.2 and 7.6 Gy, LD_60-80/60_). In order to diminish needless pain and suffering, the animals were assessed by a board-certified veterinarian and selected for early euthanasia as health declined. Animals that met the appropriate criteria were euthanized according to the American Veterinary Medical Association (AVMA) guidelines, as described earlier ^21^. The euthanasia process began with sedation via a Ketamine hydrochloride (5 – 15 mg/kg, *im*) injection. When the proper sedation plane was reached, the animals were euthanized intravenously using sodium pentobarbital (> 100 mg/kg, Euthasol, Virbac AH, Inc, Fort Worth, TX). Death was confirmed by the cessation of breathing, pulse, and heartbeat.

### Sample preparation and serum metabolomics using UPLC QTOF analysis

The sequence of samples was randomized prior to sample preparation for LC–MS analysis to avoid any bias. The sample extracts were analyzed on an Acquity UPLC coupled to a Xevo G2 QTOF-MS (Waters Corporation, Milford, MA). Each sample analyzed was 2 μL. For metabolomics acquisition, each sample was injected onto an Acquity UPLC BEH C18, 130 Å, 1.7 µm, 2.1 mm × 50 mm column maintained at 40 ℃. For lipidomics acquisition, the sample was injected onto a CSH C18, 130 Å, 1.7 µm, 2.1 mm × 100 mm column and maintained at 65 ℃. Detailed methods for metabolite extraction and LC–MS analysis using NHP samples are described earlier^14,22^. It is important to note that the identified metabolites discussed herein are putative and were determined by matching observed m/z (mass to charge) values within 0.01 Da per charge in the METLIN database (Scripps Research, La Jolla, CA)^23^.

### Statistical analysis

Statistical analysis was performed to evaluate the effects of 7.2 or 7.6 Gy total-body radiation on serum metabolite abundances, represented by normalized intensity units, in NHP serum profiles. Data was obtained from both the electrospray positive and negative modes, and was analyzed separately after internal standard and Quality Control-based Robust LOESS Signal Correction (QC-RLSC) normalization. A comprehensive list of all metabolites screened for in this study can be viewed in Supplementary Table 1. Independent (unpaired) non-parametric statistical tests of normalized intensities at different collection time points were performed separately using Mann–Whitney U tests for the 7.2 and 7.6 Gy dose group data, and these results can be viewed in Supplementary Tables 2 and 3, respectively. Supplementary Table 4 offers a comprehensive view of metabolomics data across all groups and time points. The nonparametric method is used to evaluate the distributional equality between two distinct populations, and was specifically chosen because the data does not follow normal distributions. Features exhibiting statistically significant deviations at a significance level of 0.05 (p-value < 0.05) were subsequently subjected to a false discovery rate (FDR) correction for multiple comparisons to reduce the risk of false positives, with the threshold also set at 0.05^24,25^. Only those features with FDR < 0.05 were considered further. This process yielded 895 putatively identified features of interest.

## Results

**Fig. 2:**
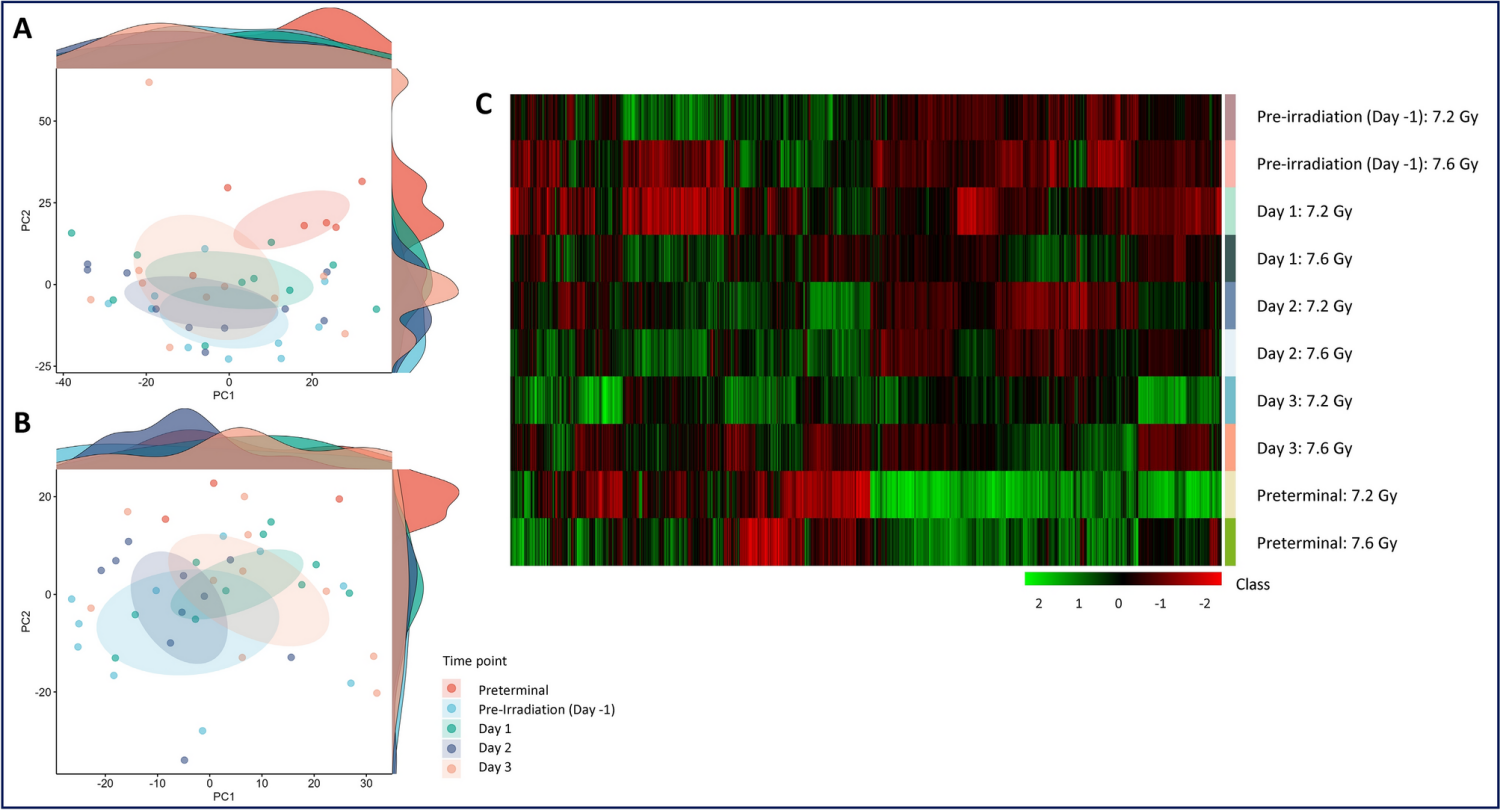
Principal Component Analysis (PCA) plots displaying the metabolomic distribution in the pre-irradiation, post-irradiation, and preterminal samples for 7.2 Gy irradiation (Panel **A**) or 7.6 Gy (Panel **B**). The heatmap (Panel **C**) similarly shows the divergence of the metabolomic profiles at the various sample timepoints.

The goal of this study was to investigate the metabolomic changes in serum of animals exposed to either 7.2 or 7.6 Gy total-body radiation by assessing differences in samples collected immediately prior to euthanasia (preterminal) compared to the pre-irradiation and post-irradiation time points. Overall, metabolomic changes after exposure to 7.2 and 7.6 Gy irradiation were found to be similar, with the PCA plots (Fig. 2, Panels A and B) providing an overall view of the variance within and across the radiation groups. Minimal differences were expected in terms of metabolic pathways affected due to the proximity in doses; however, a slightly greater degree of dysregulation was expected in the 7.6 Gy cohort. Dysregulation was typically greatest at around day 2 post-irradiation for the pre-irradiation vs. post-irradiation comparisons for 7.2 and 7.6 Gy, as well as the 7.6 Gy preterminal vs. post-irradiation comparison. The 7.2 Gy pre-irradiation vs. post-irradiation comparison was unique in that dysregulation peaked early at day 1 rather than day 2, and the majority of metabolites were significantly upregulated in the day 1 comparison rather than significantly downregulated, as in the other comparisons. Additionally, dysregulation was greater in the post-irradiation vs. preterminal comparisons than the pre-irradiation vs. post-irradiation comparisons in both the 7.2 and 7.6 Gy cohorts (Tables 1 and 2, respectively). A heat map was also constructed to visualize the overall effects of 7.2 and 7.6 Gy on metabolomic profiles (Fig. 2, panel C), which clearly indicates a strong preterminal signature that is characterized by an amplification of metabolomic dysregulation in certain pathways.

**Table 1:** Statistical analyses identified a number of putative dysregulated metabolites for each study group comparison in NHPs exposed to 7.2 Gy total-body irradiation**.**

Comparison	Total significant metabolites (*p* ≤ 0.05)	Metabolites (↑ | ↓)	Total significant metabolites (*FDR corrected P* ≤ 0.05)	Metabolites (↑ | ↓)
Pre-Irradiation (Day -1) vs. Day 1	991	792 | 199	207	125 | 82
Pre-Irradiation (Day -1) vs. Day 2	380	157 | 223	33	3 | 30
Pre-Irradiation (Day -1) vs. Day 3	217	44 | 173	16	0 | 16
Day 1 vs. Preterminal	1697	123 | 1574	1299	61 | 1238
Day 2 vs. Preterminal	1732	415 | 1317	1314	257 | 1057
Day 3 vs. Preterminal	1099	342 | 757	615	169 | 446

The comparative analysis was performed using the Mann-Whitney U Test, which is a nonparametric model.

*FDR* false discovery rate.

**Table 2:** Statistical analyses identified a number of putative dysregulated metabolites for each study group comparison in NHPs exposed to 7.6 Gy total-body irradiation.

Comparison	Total significant metabolites (*p* ≤ 0.05)	Metabolites (↑ | ↓)	Total significant metabolites (*FDR corrected P* ≤ 0.05)	Metabolites (↑ | ↓)
Pre-Irradiation (Day -1) vs. Day 1	680	153 | 527	71	34 | 37
Pre-Irradiation (Day -1) vs. Day 2	654	145 | 509	179	27 | 152
Pre-Irradiation (Day -1) vs. Day 3	681	188 | 493	72	14 | 58
Day 1 vs. Preterminal	1310	250 | 1060	842	139 | 703
Day 2 vs. Preterminal	1868	531 | 1337	1137	304 | 833
Day 3 vs. Preterminal	1354	199 | 1155	1053	161 | 892

The comparative analysis was performed using the Mann-Whitney U Test, which is a nonparametric model.

*FDR* false discovery rate.

### Both 7.2 and 7.6 Gy irradiation induced significant metabolomic dysregulation

**Fig. 3:**
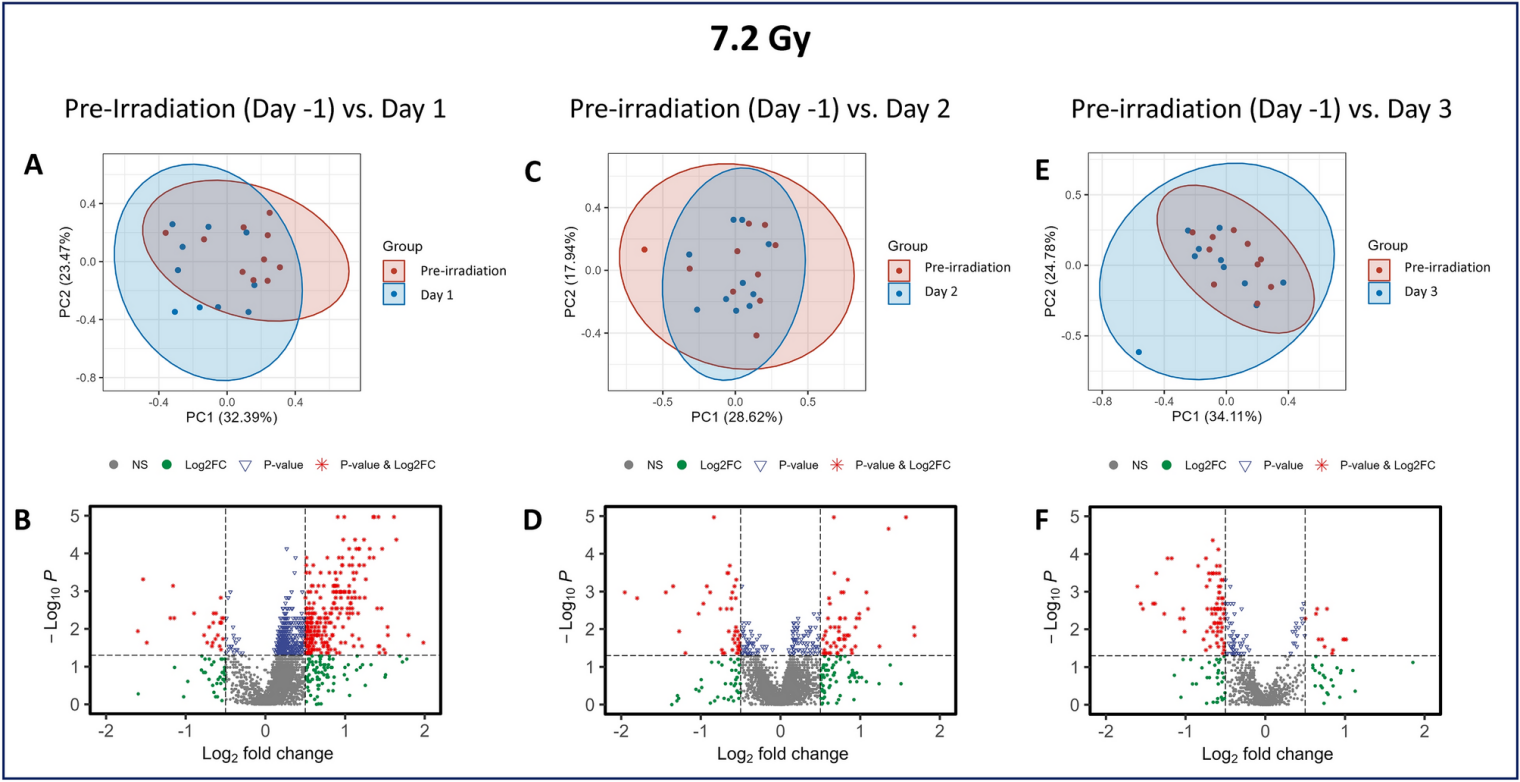
PCA and volcano plots demonstrating the metabolic differences between Day 1 (**A, B**), Day 2 (**C, D**), and Day 3 (**E, F**) to the pre-irradiation standard in NHPs exposed to 7.2 Gy irradiation.

**Fig. 4:**
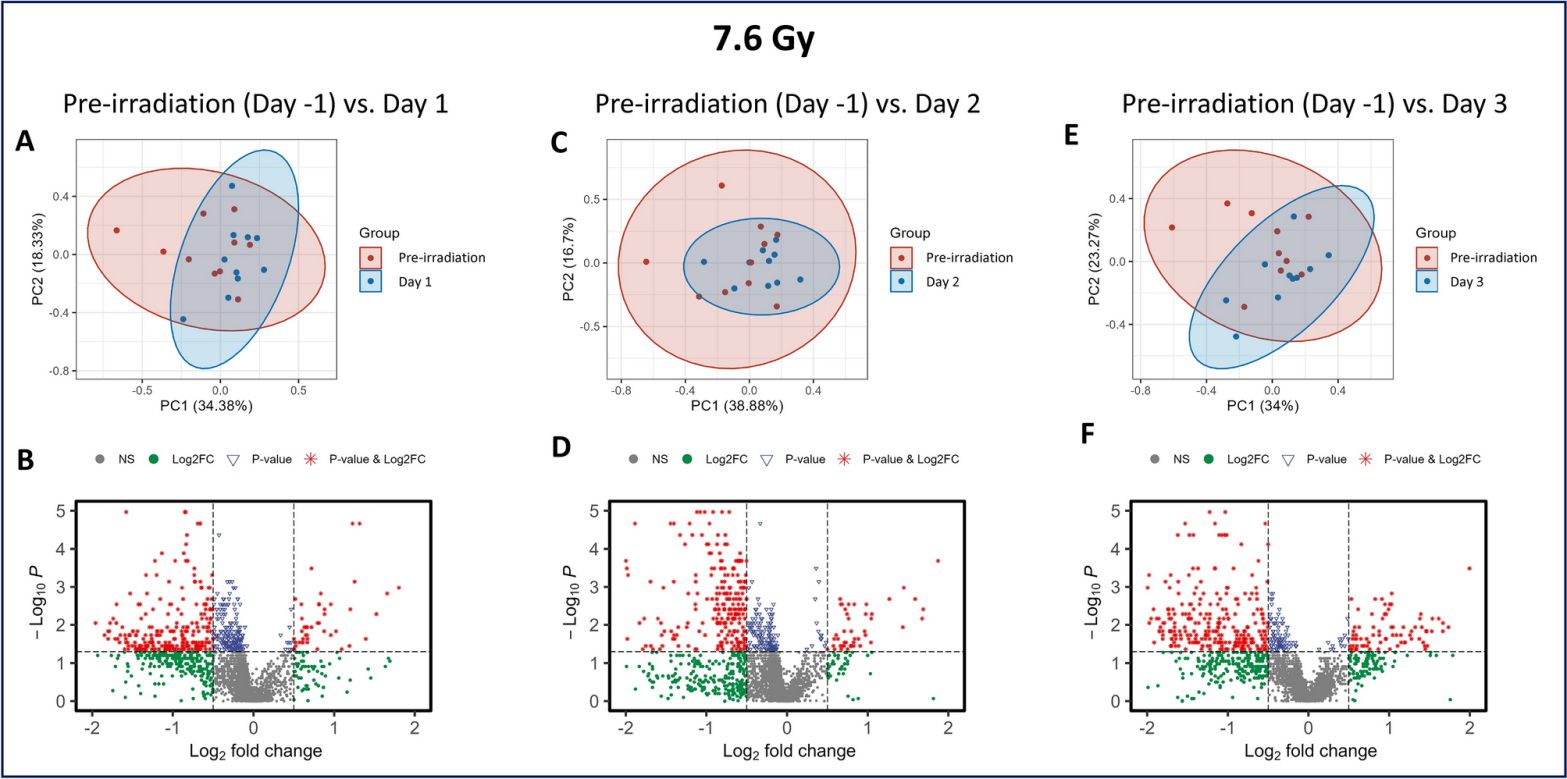
PCA and volcano plots demonstrating the metabolic differences between Day 1 (**A, B**), Day 2 (**C, D**), and Day 3 (**E, F**) to the pre-irradiation standard in NHPs exposed to 7.6 Gy irradiation.

A comparative assessment between the 7.2 Gy and 7.6 Gy cohorts revealed a high degree of similarity in their metabolic responses to ionizing radiation exposure. However, from a statistical standpoint, the higher dosage group presented a greater magnitude of significant metabolic alterations, suggesting a dose-dependent exacerbation in the metabolomic response. In the PCA plots, clear distinctions in the normalized feature intensities appear for pre-irradiation compared to post-irradiation in both the 7.2 and 7.6 Gy cohorts, indicating distinct differences between these groups (Figs. 3 and 4, Panels A, C, and E, respectively). Many similarities were noted between the two radiation doses including clear separation between the post-irradiation groups and the preterminal group. Metabolites were mostly downregulated in the time points following irradiation in both the 7.2 and 7.6 Gy cohorts (Figs. 3 and 4, panels B, D, and F, respectively).

**Fig. 5:**
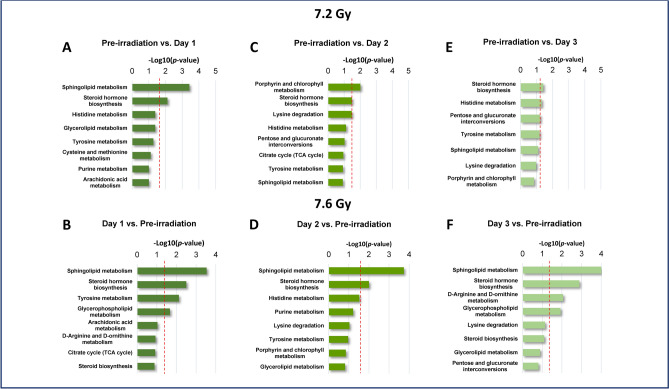
Metabolomic pathways with the most dysregulation for Day 1 (**A**), Day 2 (**C**), and Day 3 (**E**) in comparison to the pre-irradiation standards in animals exposed to 7.2 Gy, and Day 1 (**B**), Day 2 (**D**), and Day 3 (**F**) in comparison to preterminal samples in NHPs exposed to 7.6 Gy total-body irradiation.

Some of the unique alterations in the 7.2 Gy cohort included arabitol; 2,3-Diphospho-D-glyceric acid; 4-(2-Aminophenyl)-2,4-dioxobutanoic acid; and 5-Hydroxyindoleacetic acid, which were significantly downregulated in all pre-irradiation vs. post-irradiation comparisons (Table 3); while in the 7.6 Gy cohort, metabolites including trigonelline, proline betaine, and 4-Hydroxy-4-(3-pyridyl)-butanoic acid were significantly downregulated (Table 4). Pathways including sphingolipid metabolism, steroid hormone biosynthesis, and histidine metabolism, among others, were notably dysregulated in response to 7.2 Gy irradiation (Fig. 5, Panels A, C, and E). One pathway stood out as unique, namely the porphyrin and chlorophyll metabolism pathway, as it was only significant when comparing pre-irradiation to day 2 post-irradiation. As for the 7.6 Gy cohort, the same three notable pathways were significantly dysregulated by radiation (Fig. 5, Panels B, D, and F). Comprehensive pathway analysis results comparing the pre-irradiation time point to post-irradiation time points can be viewed for the 7.2 and 7.6 Gy cohorts in Supplementary Tables 5 and 6, respectively.

**Table 3:** Downregulated serum metabolites after 7.2 Gy total-body exposure.

KEGG	Name	Comparison	P-value	FDR	Fold change	Log_2_FC
C00532	Arabitol	Day -1 (Pre-Irradiation) vs. Day 1	< 0.001	< 0.05	0.488	− 1.034
		Day -1 (Pre-Irradiation) vs. Day 2	< 0.001	< 0.05	0.443	− 1.174
		Day -1 (Pre-Irradiation) vs. Day 3	< 0.001	< 0.01	0.289	− 1.789
C01159	2,3-Diphospho-D-glyceric acid	Day -1 (Pre-Irradiation) vs. Day 1	< 0.01	< 0.01	0.738	− 0.439
		Day -1 (Pre-Irradiation) vs. Day 2	< 0.001	< 0.01	0.561	− 0.835
		Day -1 (Pre-Irradiation) vs. Day 3	< 0.001	< 0.05	0.500	− 0.999
C01252	4-(2-Aminophenyl)-2,4-dioxobutanoic acid	Day -1 (Pre-Irradiation) vs. Day 1	< 0.001	< 0.05	0.472	− 1.082
		Day -1 (Pre-Irradiation) vs. Day 2	< 0.001	< 0.05	0.428	− 1.225
		Day -1 (Pre-Irradiation) vs. Day 3	< 0.001	< 0.01	0.295	− 1.762
C05635	5-Hydroxyindoleacetic acid	Day -1 (Pre-Irradiation) vs. Day 1	< 0.01	< 0.05	0.402	− 1.314
		Day -1 (Pre-Irradiation) vs. Day 2	< 0.001	< 0.05	0.230	− 2.119
		Day -1 (Pre-Irradiation) vs. Day 3	< 0.001	< 0.01	0.206	− 2.277

*KEGG* kyoto encyclopedia of genes and genomes, *FDR* false discovery rate, *FC* fold change.

**Table 4:** Downregulated serum metabolites after 7.6 Gy total-body exposure.

KEGG	Name	Comparison	P-value	FDR	Fold change	Log_2_FC
C01004	Trigonelline	Day -1 (Pre-Irradiation) vs. Day 1	< 0.001	< 0.05	0.55	− 0.864
		Day -1 (Pre-Irradiation) vs. Day 2	< 0.001	< 0.01	0.474	− 1.077
		Day -1 (Pre-Irradiation) vs. Day 3	< 0.001	< 0.01	0.475	− 1.075
C10172	Proline betaine	Day -1 (Pre-Irradiation) vs. Day 1	< 0.001	< 0.05	0.429	− 1.221
		Day -1 (Pre-Irradiation) vs. Day 2	< 0.001	< 0.01	0.368	− 1.443
		Day -1 (Pre-Irradiation) vs. Day 3	< 0.001	< 0.05	0.446	− 1.163
C19579	4-Hydroxy-4-(3-pyridyl)-butanoic acid	Day -1 (Pre-Irradiation) vs. Day 1	< 0.001	< 0.05	0.569	− 0.814
		Day -1 (Pre-Irradiation) vs. Day 2	< 0.001	< 0.01	0.493	− 1.020
		Day -1 (Pre-Irradiation) vs. Day 3	< 0.001	< 0.05	0.561	− 0.834

*KEGG* Kyoto Encyclopedia of Genes and Genomes, *FDR* false discovery rate, *FC* fold change.

### The preterminal state was characterized by an amplification of metabolomic dysregulation in metabolites related to sphingolipid metabolism

**Fig. 6:**
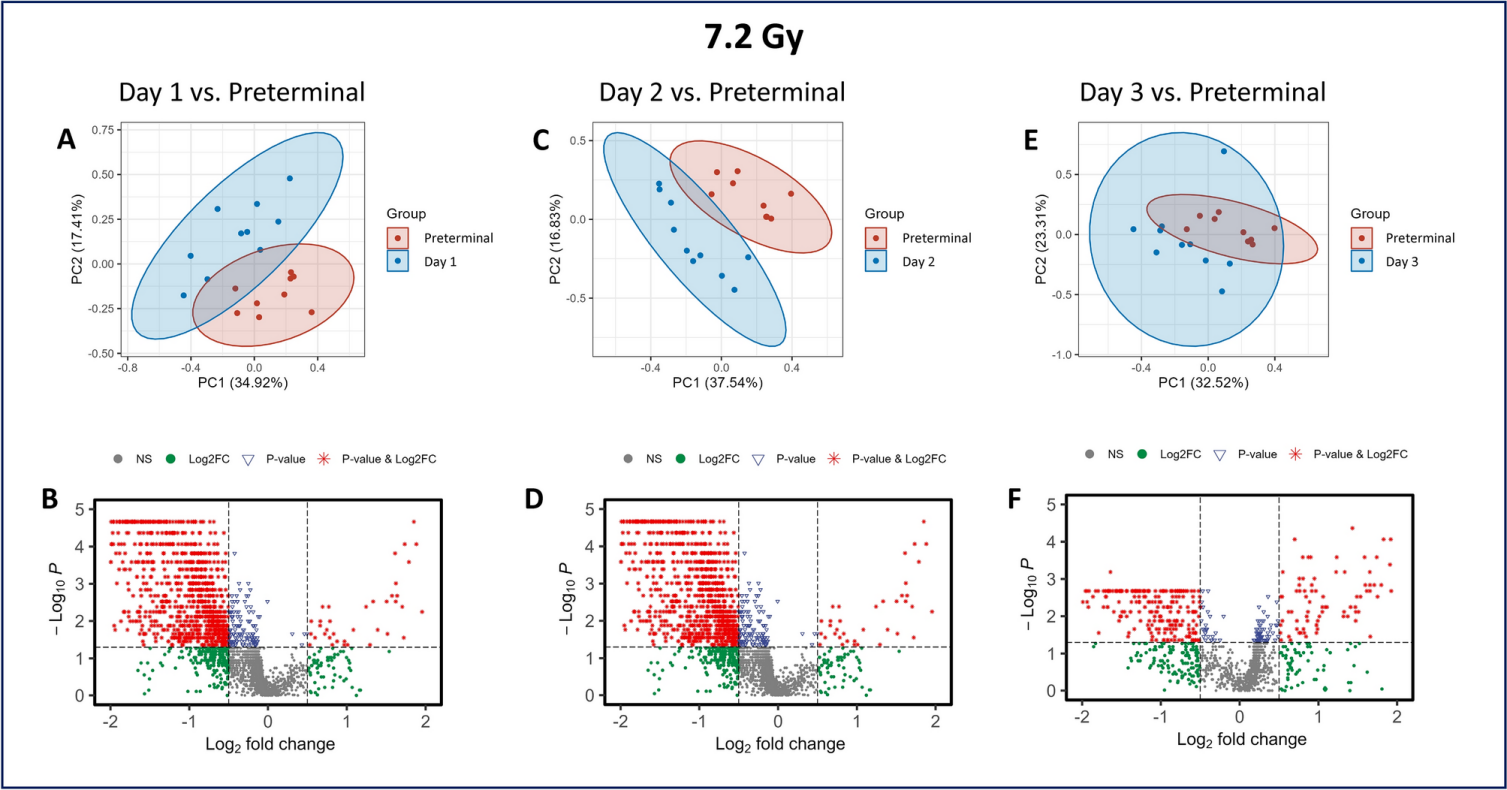
PCA and volcano plots demonstrating the metabolic differences between Day 1 (**A, B**), Day 2 (**C, D**), and Day 3 (**E, F**) to the preterminal stages in NHPs exposed to 7.2 Gy total-body irradiation.

**Fig.7:**
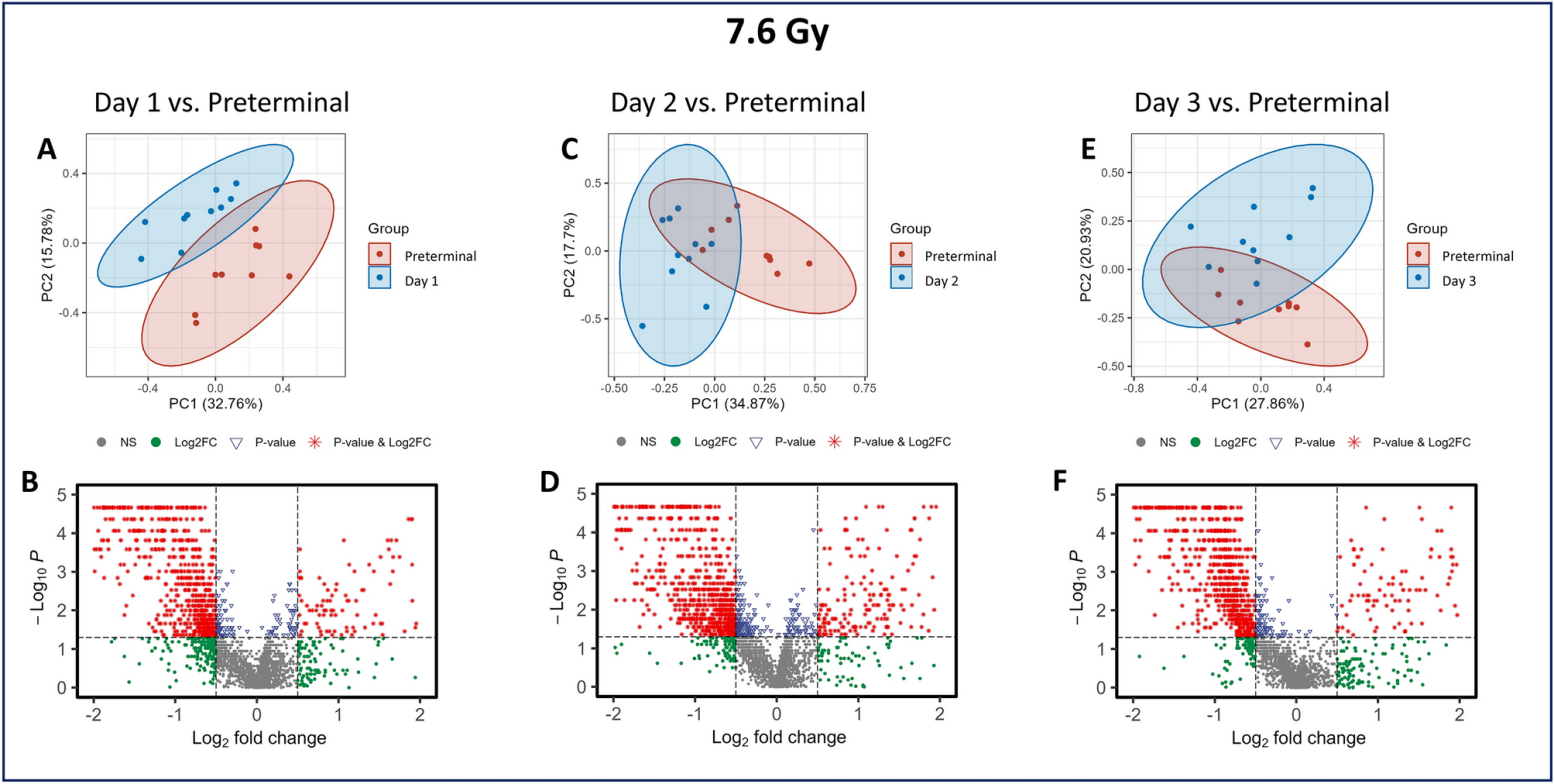
PCA and volcano plots demonstrating the metabolic differences between Day 1 (**A, B**), Day 2 (**C, D**), and Day 3 (**E, F**) to the preterminal stages in NHPs exposed to 7.6 Gy total-body irradiation.

Several metabolites and related pathways were commonly dysregulated by radiation exposure in both the 7.2 and 7.6 Gy cohorts; however, increased significance in certain metabolites was noted in the preterminal comparisons, suggesting that an exaggeration of expression of radiation-induced changes is characteristic of the preterminal state (Figs. 6 and 7, Panels A, C, and E, respectively). In the 7.2 Gy cohort, four metabolites, namely 7-Ketodeoxycholic acid, bilirubin, urobilin, and glycine appear in higher concentrations in the preterminal NHP samples for the 7.2 Gy exposure compared to all other post-irradiation time points tested. These upregulated metabolites are detailed in Table 5. In animals exposed to 7.6 Gy, four metabolites that were significantly altered in response to radiation exposure are pipecolic acid, N-Acetyl-b-glucosaminylamine, glycine, and 5-Hydroxyindoleacetic acid (Table 6). A few metabolites including methylimidazoleacetic acid, 2,3-Diphospho-D-glyceric acid, methylpentanal, pyridoxic acid, dihydrocortisol, and hydroxymandelic acid were significant when comparing the pre-irradiation time points to the post-irradiation time points in both the 7.2 and 7.6 Gy cohorts; however, an increased expression of these metabolites was noted in the pre-irradiation vs. preterminal comparisons. Volcano plots were also constructed to compare overall directionality of metabolites in post-irradiation vs. preterminal comparisons, which revealed a majority downregulation in significant metabolites for all comparisons (Figs. 6 and 7, Panels B, D, and F for the 7.2 and 7.6 Gy cohorts, respectively).

**Table 5:** Up and downregulated serum metabolites after 7.2 Gy total-body exposure in preterminal NHPs.

KEGG	Name	Comparison	P-value	FDR	Fold change	Log_2_FC
C04643	7-Ketodeoxycholic acid	Day 1 vs. Preterminal	< 0.001	< 0.001	0.098	− 3.345
		Day 2 vs. Preterminal	< 0.001	< 0.01	0.321	− 1.639
		Day 3 vs. Preterminal	< 0.01	< 0.05	0.293	− 1.770
C00486	Bilirubin	Day 1 vs. Preterminal	< 0.05	< 0.05	1.678	0.747
		Day 2 vs. Preterminal	< 0.05	< 0.05	1.687	0.754
		Day 3 vs. Preterminal	< 0.01	< 0.05	1.732	0.792
C05793	Urobilin	Day 1 vs. Preterminal	< 0.001	< 0.001	0.418	− 1.259
		Day 2 vs. Preterminal	< 0.001	< 0.001	0.336	− 1.574
		Day 3 vs. Preterminal	< 0.01	< 0.05	0.320	− 1.643
C01324	Glycine	Day 1 vs. Preterminal	< 0.01	< 0.05	2.522	1.335
		Day 2 vs. Preterminal	< 0.01	< 0.05	2.629	1.394
		Day 3 vs. Preterminal	< 0.001	< 0.01	5.039	2.333

*KEGG* Kyoto Encyclopedia of Genes and Genomes, *FDR* false discovery rate, *FC* fold change.

**Table 6:** Up and downregulated serum metabolites after 7.6 Gy total-body exposure in preterminal NHPs.

KEGG	Name	Comparison	P-value	FDR	Fold change	Log_2_FC
C00408	Pipecolic Acid	Day 1 vs. Preterminal	< 0.001	< 0.01	2.095	1.067
		Day 2 vs. Preterminal	< 0.001	< 0.001	2.461	1.299
		Day 3 vs. Preterminal	< 0.001	< 0.001	2.111	1.078
C01239	N-Acetyl-b-glucosaminylamine	Day 1 vs. Preterminal	< 0.001	< 0.001	0.138	-2.854
		Day 2 vs. Preterminal	< 0.01	< 0.01	0.291	-1.783
		Day 3 vs. Preterminal	< 0.001	< 0.001	0.191	-2.386
C01324	Glycine	Day 1 vs. Preterminal	< 0.001	< 0.01	4.844	2.276
		Day 2 vs. Preterminal	< 0.001	< 0.01	6.195	2.631
		Day 3 vs. Preterminal	< 0.001	< 0.001	5.524	2.466
C05635	5-Hydroxyindoleacetic acid	Day 1 vs. Preterminal	< 0.001	< 0.001	0.167	-2.578
		Day 2 vs. Preterminal	< 0.001	< 0.001	0.304	-1.717
		Day 3 vs. Preterminal	< 0.01	< 0.01	0.310	-1.688

*KEGG* Kyoto Encyclopedia of Genes and Genomes, *FDR* false discovery rate, *FC* fold change.

**Fig. 8:**
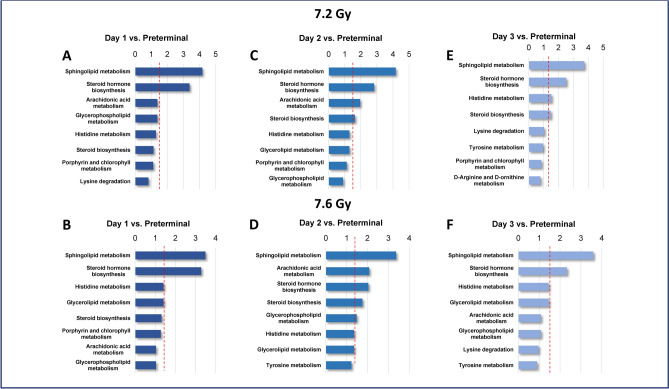
Metabolomic pathways with the most dysregulation for Day 1 (**A**), Day 2 (**C**), and Day 3 (**E**) in comparison to the preterminal samples in NHPs exposed to 7.2 Gy total-body irradiation, and Day 1 (**B**), Day 2 (**D**), and Day 3 (**F**) in comparison to preterminal samples in NHPs exposed to 7.6 Gy total-body irradiation.

Interestingly, the same pathways that were significantly dysregulated in the 7.2 Gy pre-irradiation vs. post-irradiation comparisons were also significantly dysregulated in the post-irradiation vs. preterminal comparisons, particularly in the sphingolipid metabolism and steroid hormone biosynthesis pathways, as indicated in Fig. 8, Panels A, C, and E. As for the 7.6 Gy cohort, dysregulation in both comparisons was consistent, with minimal differences between comparisons. Sphingolipid metabolism and steroid hormone biosynthesis pathways were significantly dysregulated, along with the glycerophospholipid metabolism pathway (Fig. 8, Panels B, D, and F). An exaggerated response was noted in the preterminal comparisons in both radiation cohorts, characterized by a higher degree of significance in these metabolic pathways. Comprehensive pathway analysis results for the post-irradiation vs. preterminal comparisons can be viewed for the 7.2 and 7.6 Gy cohorts in Supplementary Tables 7 and 8, respectively.

### Biochemical changes associated with radiation exposure and the preterminal effect

**Fig. 9:**
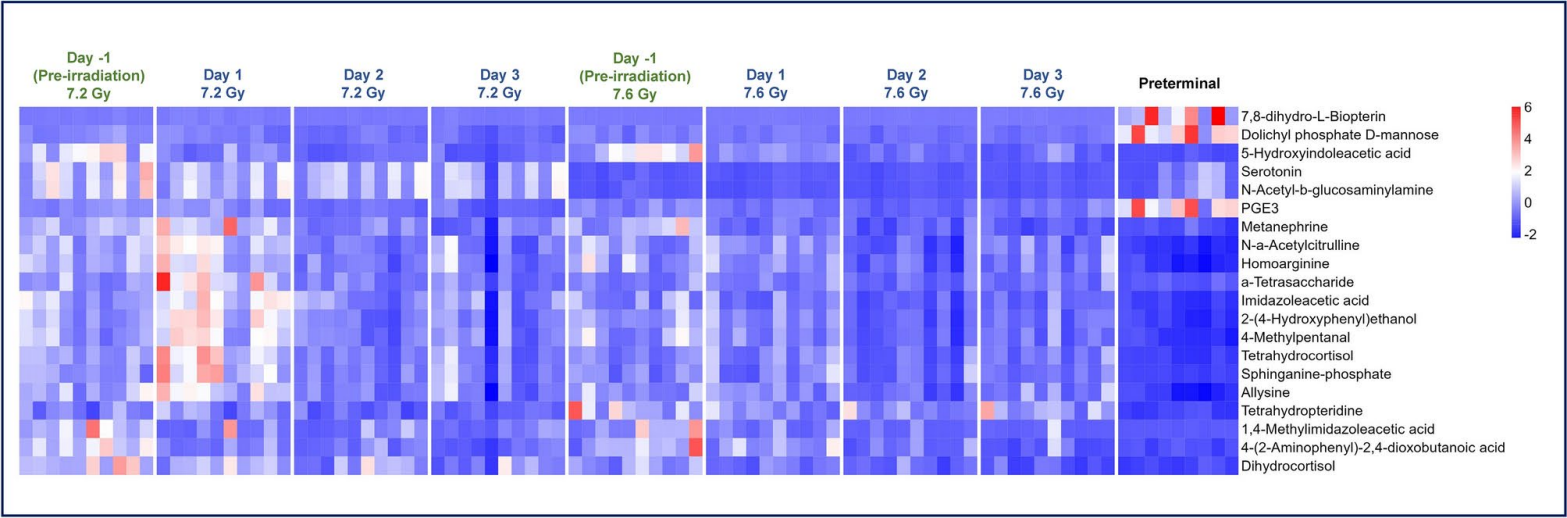
A heatmap illustrating the most significantly altered metabolites over the course of the study.

**Fig. 10:**
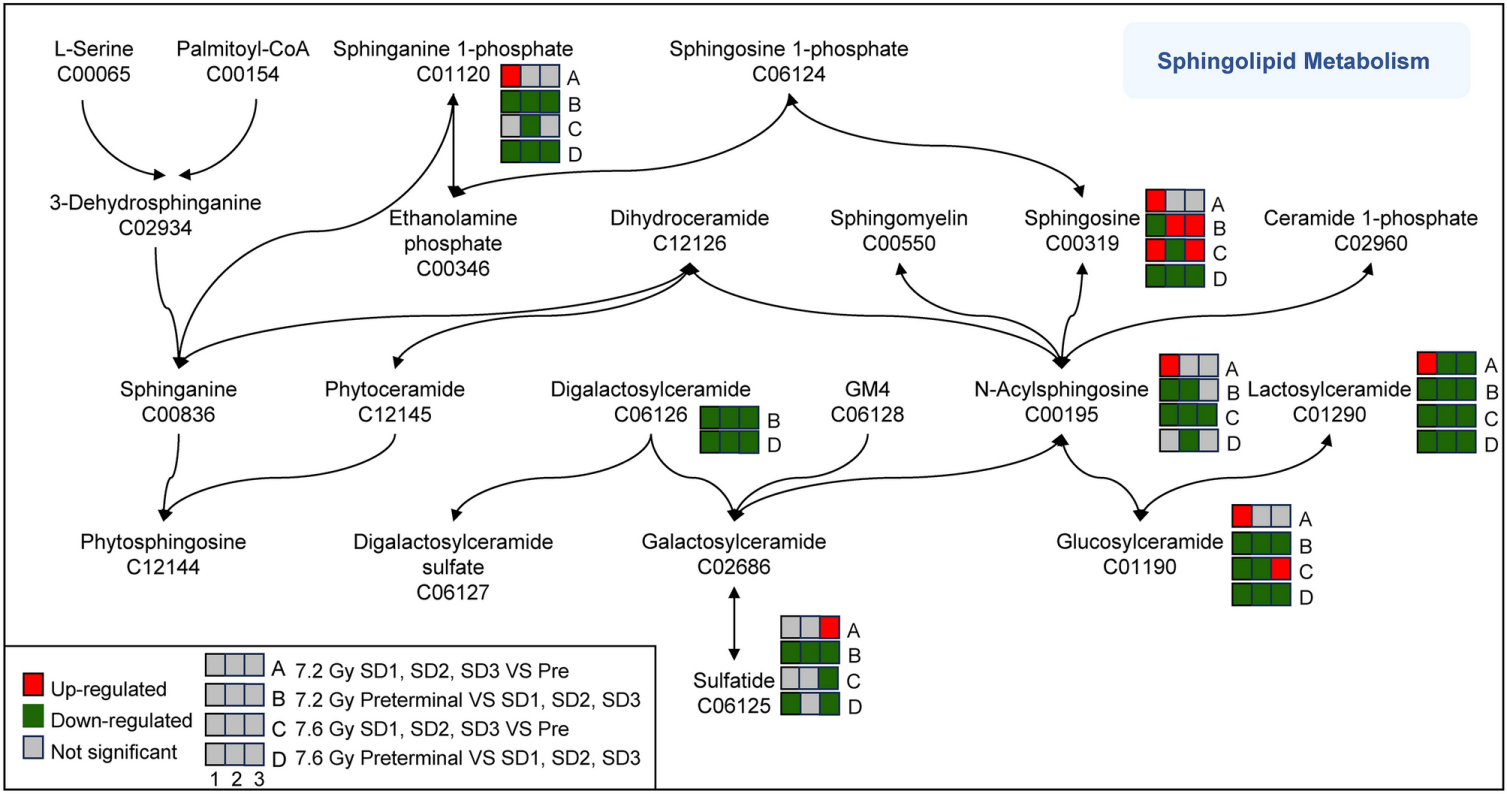
Comprehensive analysis identified consistent dysregulation in the sphingolipid metabolism pathway and the associated metabolites across both 7.2 Gy and 7.6 Gy radiation groups.

Comprehensive analysis was performed comparing the post-irradiation and preterminal signatures to determine similarities and differences in metabolomic responses. Overall, significantly dysregulated metabolites were noted in both comparisons. The heat map in Fig. 9 illustrates some of the most significantly dysregulated metabolites. Consistent perturbations in pathways such as sphingolipid metabolism, steroid hormone biosynthesis, and glycerophospholipid metabolism across both 7.2 Gy and 7.6 Gy radiation groups were noted. Notably, the preterminal groups, irrespective of the radiation dosage, manifested more distinct metabolic deviations than those solely influenced by radiation. This distinction was further underscored by the emergence of unique metabolic pathways, like arachidonic acid metabolism, which was exclusively significant in the preterminal groups. Upon examination of these radiation-induced metabolomic changes, a deeper dive into the metabolites associated with the sphingolipid metabolism pathway revealed intricate patterns of modulation post-irradiation. Specifically, metabolites such as sphinganine phosphate, digalactosylceramide, sulfatide, acylsphingosine, glucosylceramide, and lactosylceramide predominantly exhibit a downregulated trend in both the post-irradiation and preterminal groups (Fig. 10). This consistent downregulation across groups suggests a potential systemic signature that can utilized to predict the preterminal state as a consequence of acute radiation exposure.

## Discussion

Metabolomic changes induced by lethal doses of radiation exposure have been extensively studied in our laboratory using a variety of matrices in murine and NHP model systems. We have examined altered metabolites in both biofluids (serum/plasma) and tissue samples using murine ^26–28^ and NHP ^14,22,29–32^ models that were exposed to ^60^Co total-body γ-radiation, and also in NHPs with partial-body exposure with LINAC-derived photon radiation^22^. Various radiation MCMs that are being developed for prophylactic or mitigative use were administered to assess the effects on metabolites, including Ex-Rad^19^, BIO 300^33,34^, BBT-059^35^, amifostine^26–28^, and GT3^22,36,37^. The Ex-Rad^19^, GT3^22,36,37^, BBT-059^35^, and BIO 300^33,34^ studies were performed with an NHP model, while the amifostine^26–28^ studies were performed in a murine model. Recently, using transcriptomic analysis, it has been demonstrated that in a prefinal/preterminal stage (samples collected from moribund animals just prior to death) after lethal radiation exposure, the ribosome/transcriptome status remains present in 60 – 67% of animals, but in general, the whole transcriptome activity appears silenced and cannot be used for the purpose of biodosimetry^38^. We have conducted a proteomic study using preterminal samples of irradiated NHPs and demonstrated that the expression of thrombospondin and integrin alpha correlated in peripheral blood with the preterminal stage. Radiation exposure induced a temporal response in which some features were upregulated while others were downregulated. These significantly altered features varied from pre-exposure levels by as much as tenfold^39^. Ultimately, the goal of our research is to determine whether radiation-induced changes in certain metabolites from easily attainable biofluids can be consistently identified as indicators of a declining health state^38^. Not only will this research assist in radiation countermeasure development, it will also be invaluable to the treatment of radiation-exposed patients.

In the current study, animals were exposed to either 7.2 or 7.6 Gy ^60^Co gamma-irradiation and metabolomic profiles in serum samples collected pre-irradiation (day -1) were compared to serum samples collected post-irradiation (days 1, 2, or 3) or to serum samples collected immediately prior to euthanasia in moribund animals (preterminal). The degree to which radiation dysregulated metabolic pathways was expected to be substantial, as has been observed in other metabolomic studies utilizing a similar radiation dose^14,30,32^. Overall, both the 7.2 and 7.6 Gy cohorts showed a high degree of metabolic dysregulation, but the 7.6 Gy cohort experienced slightly greater significance in the number of dysregulated metabolites, indicating the possibility of a radiation dose dependent metabolic exacerbation. One finding that remained consistent between both cohorts is that greater metabolic disruption was present in the preterminal groups compared to all of the other post-irradiation timepoints. It is important to note that the significant metabolic changes observed in the preterminal state may reflect not only the effects of radiation exposure but also the natural progression toward death. In this study, both 7.2 and 7.6 Gy radiation doses induced significant metabolomic dysregulation, and we observed further exacerbation of these changes in the preterminal state. While radiation-induced metabolic dysregulation is a key factor, additional changes observed in the preterminal state suggest a physiological response that goes beyond the effects of radiation alone. These may be reflective of the critical condition of animals as they approach the terminal state. Therefore, the metabolic differences between post-irradiation and preterminal states are likely a combination of radiation-induced damage and the declining health of preterminal animals.

The current study was performed to validate the results of a previous metabolomic study performed with 7.2 Gy total-body radiation^14^. For the preliminary metabolomics study using moribund NHPs exposed to 7.2 Gy total-body radiation, we reported a greater degree of radiation-induced metabolic dysregulation in preterminal samples when compared to the post-irradiation samples tested in the study^14^. Both the glycerophospholipid metabolism and steroid hormone and biosynthesis pathways were significantly dysregulated in preterminal animals, while the glycerophospholipid metabolism was found to be dysregulated both in post-irradiation and preterminal samples. Commonalities in both dysregulated pathways and metabolites existed between the preliminary and validation studies. Both studies demonstrated significant dysregulation to lipid metabolism pathways, specifically the glycerophospholipid metabolism pathway in the preliminary study and the sphingolipid metabolism pathway in the current study, in addition to the steroid hormone biosynthesis pathway. Additionally, a few metabolites were consistently and significantly dysregulated in both the pre-irradiation vs. post-irradiation and post-irradiation vs. preterminal comparisons across both the preliminary and validation studies. Specifically, PC(18:1(9Z)/0:0)[U] / PC(18:1(9Z)/0:0)[rac] was linked to the glycerophospholipid pathway, while Lactosylceramide (d18:1/12:0) and Ganglioside GA2 (d18:1/16:0) were linked to the sphingolipid metabolism pathway. Other dysregulated metabolites common to the two studies include 5-Hydroxyindoleacetic acid, N1-acetylspermine, glycine, and iduronic acid.

Comprehensive analysis of metabolomic shifts in these preterminal groups revealed consistent disturbances in pathways such as sphingolipid metabolism, steroid hormone biosynthesis, and glycerophospholipid metabolism for both 7.2 and 7.6 radiation groups. The dysregulation of sphingolipid metabolism is consistent with known cellular and physiological responses to radiation exposure. Ionizing radiation generates reactive oxygen species (ROS) and induces oxidative stress, leading to damage in cellular membranes and contributing to lipid peroxidation. Sphingolipids, including sphingosine, play a crucial role in mediating cell survival, apoptosis, and inflammation; processes that are typically altered during radiation-induced injury. Similarly, the dysregulation of steroid hormone biosynthesis and glycerophospholipid metabolism reflects disruptions in lipid homeostasis and membrane integrity, which are essential components of cellular response to radiation damage. The metabolite sphingosine stands out as being primarily upregulated in both the post-irradiation and preterminal groups. This upregulation may indicate an accumulation of sphingosine after exposure to acute ionizing irradiation, which could have significant implications for cellular signaling and response mechanisms post-irradiation. Sphingosine was significant only in a few of the pre-irradiation vs. post-irradiation comparisons in both the 7.2 and 7.6 Gy cohorts, but was significantly downregulated in both 7.2 and 7.6 Gy post-irradiation vs. preterminal comparisons.

Lipidomic pathways, namely the sphingolipid metabolism and glycerophospholipid metabolism pathways, were consistently dysregulated in both our initial study performed with 7.2 Gy and this current validation study performed with 7.2 and 7.6 Gy. As previously mentioned, ionizing radiation is known to generate free radicals, which can accumulate in cells and contribute to dyslipidemia. Additionally, radiation causes an increase in lipid peroxidation in a radiation dose-dependent manner, indicating these pathways can be leveraged for the development of biomarkers that can accurately assess absorbed radiation doses^40,41^. Another study supports our observation by underscoring the potential therapeutic relevance of targeting sphingolipid pathways in radiation-induced injuries^42^. The patterns observed in this study, especially the distinct behavior of sphingosine, could pave the way for further investigations into the therapeutic potential of modulating sphingolipid metabolism in radiation treatments. The next steps in our research include confirming these metabolite identities in both the preliminary and validation studies, and by comparing tandem mass spectrometry fragmentation spectra to the unknown in-sample compound and a recognized standard compound.

There are some limitations of this study. In our earlier study, we collected samples on days -7, 1, 13, 25, and preterminal collection. In this study we used samples of days -1, 1, 2, 3, and preterminal collection. This is simply due to the availability of such samples. It is important to note that despite this difference in time points, we are able to confirm the findings of our earlier preliminary study^14^. Furthermore, we have used only 10 animals in each group and all animals are males. Although n = 10 is a reasonable number for an NHP study, confirmation with a larger sample size with animals of both sexes in the future will be valuable. In this study, only cobalt-60 gamma radiation source has been used. Findings of this study may need to be confirmed using other radiation sources, particularly mixed field radiation (gamma and neutron). It also needs to be pointed out that the current study includes only two total-body radiation exposures, 7.2 and 7.6 Gy. Using a broader range of radiation doses would not only further confirm our observations, but it would be essential in order to advocate for using identified metabolites as biomarkers. Similarly, partial-body exposure and the use of various levels of supportive care also need to be investigated. Additionally, the metabolite identification in this study was based on parent m/z (putative), and while this allowed us to conduct pathway analysis, we acknowledge that further LC–MS/MS validation is necessary to improve the accuracy of metabolite identification which will strengthen the pathway analysis results. In brief, the results of this study offer important insights for identification and validation of biomarkers for lethality, and the identification of such metabolomic biomarkers would be valuable for the triage of human radiation injury scenarios.

## Supplementary Information

Supplementary Tables.

## Data Availability

All relevant data are within the manuscript and its Supporting Information files.
